# Supplementary material for: Omega-3 PUFAs prevent bone impairment and bone marrow adiposity in mouse model of obesity
Source: Commun Biol. 2023 Oct 14;6:1043. doi: 10.1038/s42003-023-05407-8 (PMC10575870; doi:10.1038/s42003-023-05407-8)
Supplement: Supplementary file 2 — Supplementary information [file 42003_2023_5407_MOESM2_ESM.pdf]

SUPPLEMENTARY INFORMATION

CONTENT:

Supplementary Tables: Supplementary Table 1-4

Supplementary Figures with figure legends: Supplementary Figure S1-10

SUPPLEMENTARY TABLES

Supplementary Table 1: Macronutrient composition and energy content in the experimental diets.

	ND	HFD	HFD+F
**Lipids (% diet, wt/wt)**	2.9	31.8	32.4
**Carbohydrates (% diet, wt/wt)**	64.3	47.2	47.6
**Proteins (% diet, wt/wt)**	16.9	11.7	11.7
**Energy density (kJ/g)**	13.0	20.1	20.1
**Supplement:**			
**Epax 1050 TG (g/100g)**	0.0	0.0	5.3

Supplementary Table 2: Fatty acid composition of dietary lipids

(mol %)	**ND**	**HFD**	**HFD+F**
12:0	0.15	0.26	-
14:0	0.14	1.22	-
16:0	12.52	13.42	12.05
16:1 (n-9)	-	-	-
16:1 (n-7)	0.32	0.26	0.31
18:0	6.00	3.34	3.84
18:1 (trans)	0.55	0.42	0.45
18:1 (n-9)	25.64	31.00	28.44
18:1 (n-7)	1.59	0.65	0.87
18:2 (n-6)	46.62	47.22	41.10
18:3 (n-6)	-	-	-
20:0	0.42	0.43	0.50
18:3 (n-3)	4.89	1.24	1.24
20:1 (n-9)	0.31	0.26	0.47
20:2 (n-6)	-	-	0.14
20:3 (n-6)	-	-	-
20:4 (n-6)	-	-	0.47
20:5 (n-3)	-	-	1.65
22:4 (n-6)	-	-	0.12
22:5 (n-6)	-	-	0.48
22:5 (n-3)	-	-	0.34
22:6 (n-3)	0.45	-	6.39
SFA	23.71	19.48	18.14
MUFA	27.92	32.26	30.18
n-6 PUFA	46.86	47.34	42.35
n-3 PUFA	0.97	0.49	8.87

Fatty acid composition in total dietary lipids was determined using gas chromatography.
SFA, saturated fatty acid; MUFA, monounsaturated fatty acid. -, <0.1 %.

Supplementary Table 3: List of mouse primers

**Gene name**	**Gene Sequence 5'-3'**
36B4 F	TCCAGGCTTTGGGCATCA
36B4 R	CTTTATCAGCTGCACATCACTCAGA
Fsp27 F	ATCAGAACAGCGCAAGAAGA
Fsp27 R	CAGCTTGTACAGGTCGAAGG
Cd36 F	ATGGGCTGTGATCGGAACTG
Cd36 R	TTTGCCACGTCATCTGGGTTT
Alpl F	GCCCTCTCCAAGACATATA
Alpl R	CCATGATCACGTCGATATCC
Bmp2 F	GGGACCCGCTGTCTTCTAGT
Bmp2 R	TCAACTCAAATTCGCTGAGGAC
Col1a1 F	GGTGAACAGGGGTTCCTGG
Col1a1 R	TTCGCACCAGGTTGCCATC
Adipoq F	GACGTTACTACAACTGAAGAGC
Adipoq R	CATTCTTTTCCTGATACTGGTC
Cebpa F	AAGCCAAGAAGTCGGTGGA
Cebpa R	CAGTTCACGGCTCAGCTGTTC
Il1β F	GCAACTGTTCCTGAACTCAACT
Il1β R	ATCTTTTGGGGTCCGTCAACT
Tnfα F	CCCTCACACTCAGATCATCTTCT
Tnfα R	GCTACGACGTGGGCTACAG
p53 F	TCTTATCCGGGTGGAAGGAAA
p53 R	GGCGAAAAGTCTGCCTGTCTT
p16 F	GGGTTTTCTTGGTGAAGTTCG
p16 R	TTGCCCATCATCATCACCT
Sod2 F	CAGACCTGCCTTACGACTATGG
Sod2 R	CTCGGTGGCGTTGAGATTGTT
Hmox1 F	AGGTACACATCCAAGCCGAGA
Hmox1 R	CATCACCAGCTTAAAGCCTTCT

Pparγ2 F	GGGTCAGCTCTTGTGAATGG
Pparγ2 R	CTGATGCACTGCCTATGAGC
Oc F	TGCGCTCTGTCTCTCTGACC
Oc R	CTGTGACATCCATACTTGCAGG
p21 F	CCTGGTGATGTCCGACCTG
p21 R	CCATGAGCGCATCGCAATC
Trap F	CAGCTCCCTAGAAGATGGATTCAT
Trap R	GTCAGGAGTGGGAGCCATATG
Rankl F	AGCCGAGACTACGGCAAGTA
Rankl R	AAAGTACAGGAACAGAGCGATG
Rela F	ACTGCCGGGATGGCTACTAT
Rela R	TCTGGATTCGCTGGCTAATGG
Opg F	CCTTGCCCTGACCACTCTTAT
Opg R	CACACACTCGGTTGTGGGT
Ctsk F	AGGCAGCTAAATGCAGAGGGTACA
Ctsk R	AGCTTGCATCGATGGACACAGAGA
Ctnnb1 F	CCCAGTCCTTCACGCAAGAG
Ctnnb1 R	CATCTAGCGTCTCAGGGAACA
Vegfa F	GTACCTCCACCATGCCAAGTG
Vegfa R	TGGGACTTCTGCTCTCCTTCTG
Vcam F	GGCTCCAGACATTTACCCAGTT
Vcam R	CATGAGCTGGTCACCCTTGAA
Fas F	CTGCACCCTGACCCAGAATAC
Fas R	ACAGCCAGGAGAATCGCAGTA
Fasgl F	CAGTCCACCCCCTGAAAAAAA
Fasgl R	CCTTGAGTTGGACTTGCCTGTT
Il10 F	CTGGACAACATACTGCTAACCG
Il10 R	GGGCATCACTTCTACCAGGTAA
Il1rn F	GCTCATTGCTGGGTACTTACAA
Il1rn R	CCAGACTTGGCACAAGACAGG

Supplementary Table 4. List of primary and secondary antibodies used for western blot

**Primary antibodies**	**Catalog #**	**Company**	**Dilution**
Phospho-Akt Ser473_ Rabbit	#4058 (193H12)	Cell Signaling	1:1000
Phospho-Akt Thr308_Rabbit	#13038 (D25E6)	Cell Signaling	1:1000
Total AKT_Rabbit	#9272	Cell Signaling	1:1000
β-actin_Rabbit	#4970 (13E5)	Cell Signaling	1:1000
Phospho-NF-kB p65_Rabbit	#3031	Cell Signaling	1:1000
NF-kB p65_Rabbit	#8242 (D14E12)	Cell Signaling	1:1000
**Secondary HRP-conjugated antibodies**			
Anti-rabbit IgG, HRP-linked Antibody	#7074	Cell Signaling	1:5000

Supplementary Fig. 1

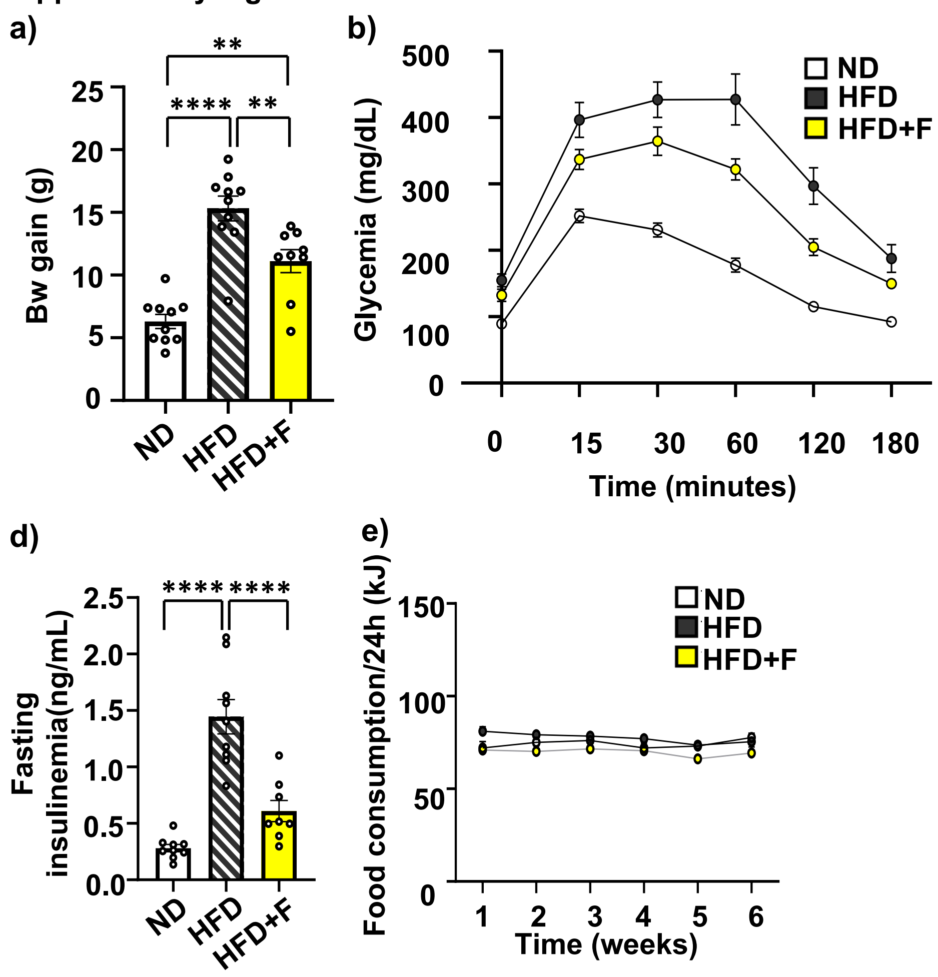

Supplementary Fig. 1 legend: Metabolic parameters of investigated mice.
(**a-d**) Omega-3 PUFAs improve metabolic parameters in HFD mice. (**a**) Body weigh gain, (**b**) measurement of intraperitoneal glucose tolerance test (GTT) in treated mice at the end of dietary intervention after overnight fasting (groups coding: white circle-ND, black circle-HFD, yellow circle-HFD+F), (**c**) area under the curve of coresponding GTT graphs; (**d**) insulinemia after overnight fasting at the end of dietary intervention in treated mice (n= 7-10). (**e**) Food intake (kJ) in treated mice during dietary intervention (n=7-10). Data are presented as mean ± SEM (n = 6-8 per group); one-way ANOVA, Tukey's multiple comparison test with * p ≤ 0.05, ** p ≤ 0.01, **** p ≤ 0.0001. (groups coding: white column-ND, black line shading-HFD, yellow column-HFD+F)

Supplementary Fig. 2

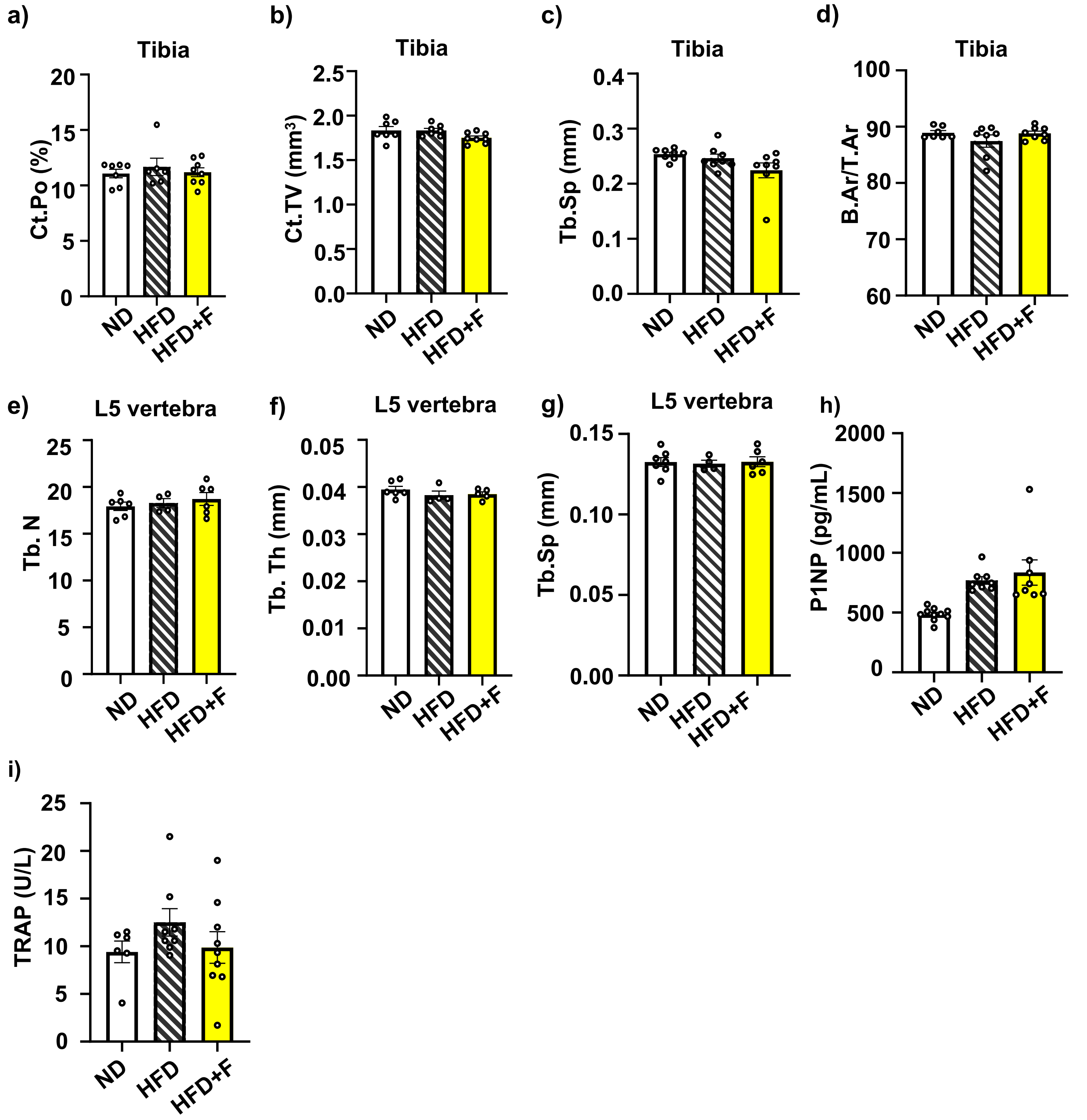

Supplementary Fig. 2 legend: Bone parameters of investigated mice.
(**a-d**) Evaluation of bone parameters in tibia: (**a**) Cortical porosity (Ct.Po), (**b**) Cortical total volume (Ct.TV); (**c**) trabecular separation (Tb.Sp) and (**d**) cortical area fraction (B.Ar/T.Ar) in treated mice (n= 7-10). (**e-g**) Evaluation of bone parameters in L5 vertebra: (**e**) Trabecular number (Tb.N), (**f**) trabecular thickness (Tb.Th), (**g**) trabecular separation (Tb.Sp). Analysis of circulating (**h**) bone formation marker P1NP (pg/mL) and (**i**) bone resorption marker TRAP (U/L). Data are presented as mean ± SEM (n = 6-8 per group); one-way ANOVA, Tukey's multiple comparison test with * $p \leq 0.05$. (groups coding: white column-ND, black line shading-HFD, yellow column-HFD+F)

Supplementary Fig. 3

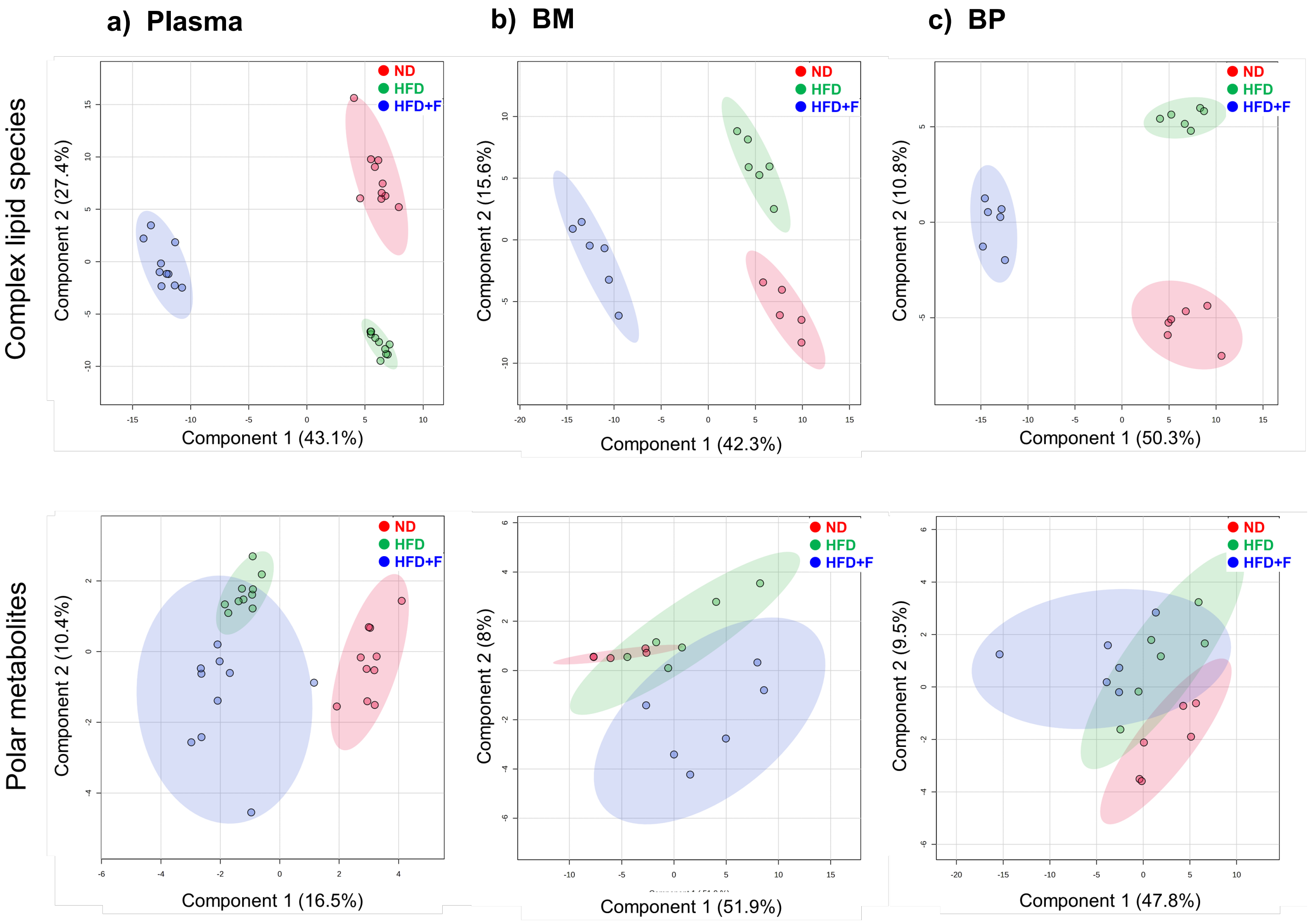

Supplementary Fig. 3 legend: Metabolomic analysis of plasma, bone marrow (BM) and bone powder samples obtained from investigated mice.
PLS-DA score plots of unique lipid species (top panels) and polar metabolites (bottom panels) for (**a**) plasma, (**b**) bone marrow, and (**c**) bone powder samples.

Supplementary Fig. 4

Plasma

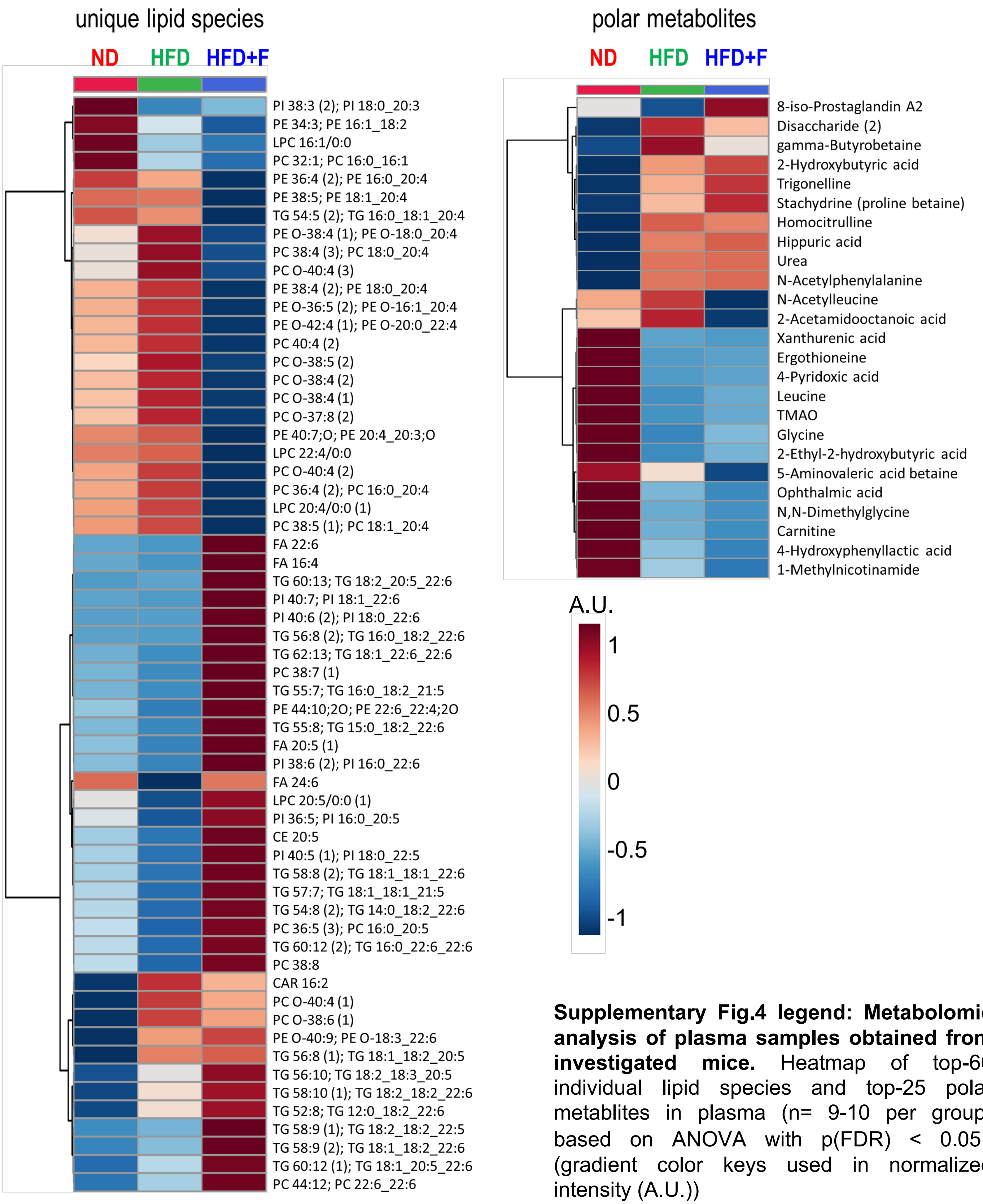

Supplementary Fig.4 legend: Metabolomic analysis of plasma samples obtained from investigated mice. Heatmap of top-60 individual lipid species and top-25 polar metablites in plasma (n= 9-10 per group) based on ANOVA with p(FDR) < 0.05,. (gradient color keys used in normalized intensity (A.U.))

Supplementary Fig. 5

BM

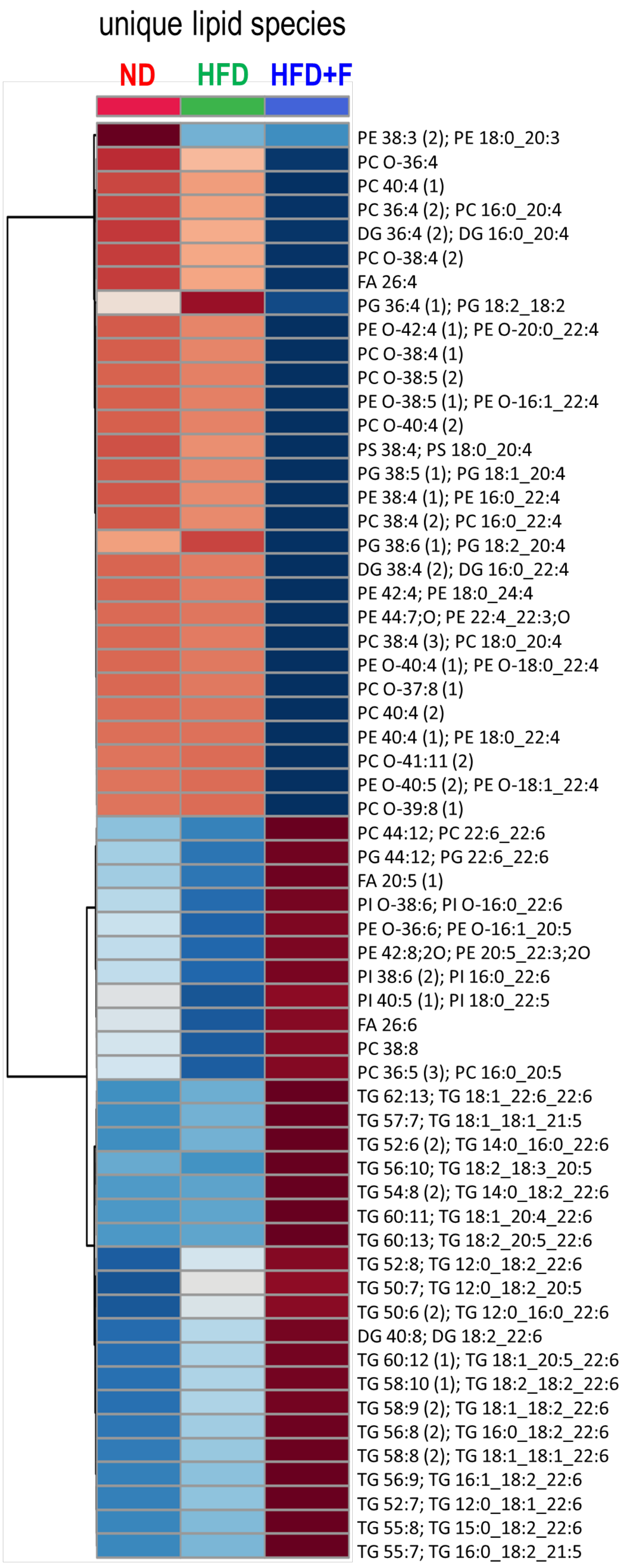

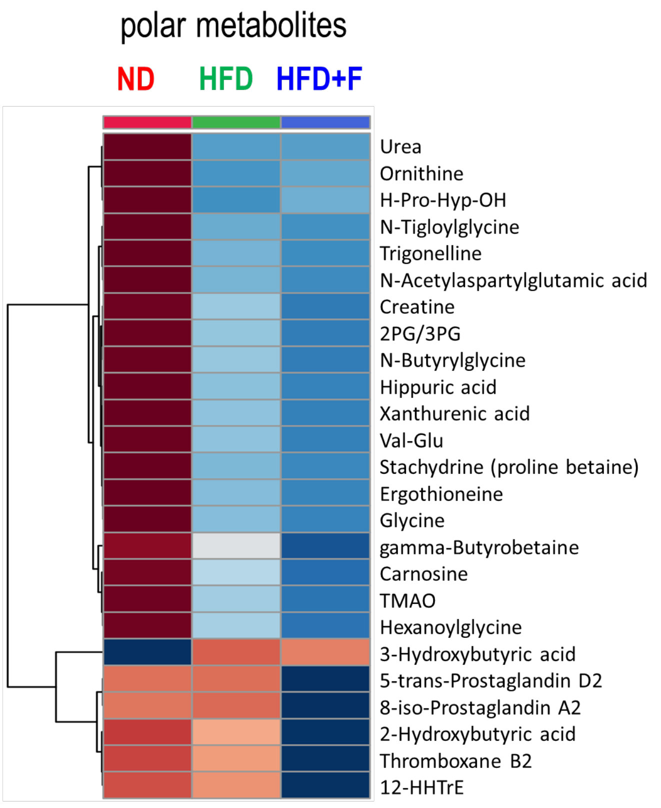

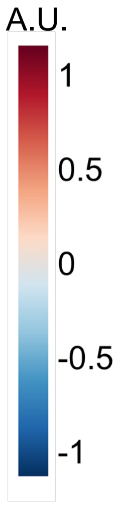

Supplementary Fig.5 legend: Metabolomic analysis of bone marrow (BM) samples obtained from investigated mice. Heatmap of top-60 individual lipid species and top-25 polar metablites in BM (n= 5-6 per group) based on ANOVA with p(FDR) < 0.05. (gradient color keys used in normalized intensity (A.U.))

Supplementary Fig. 6

BP

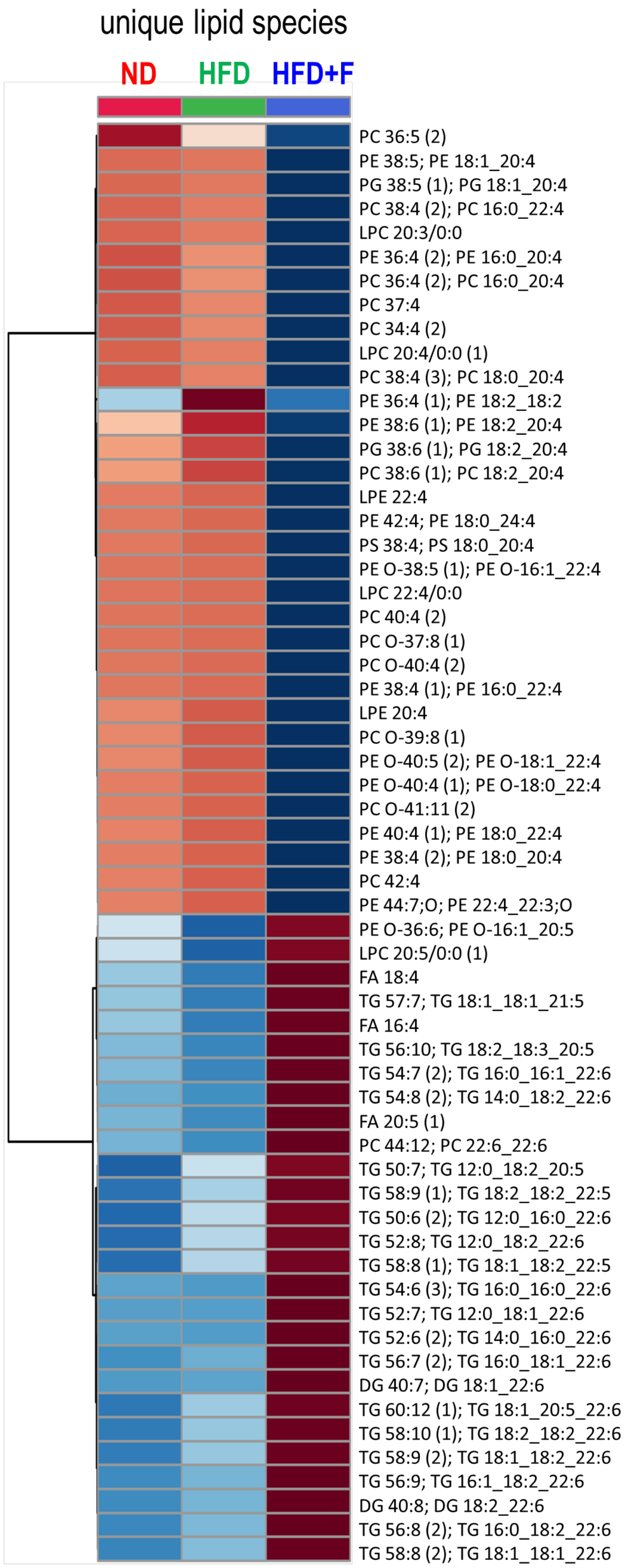

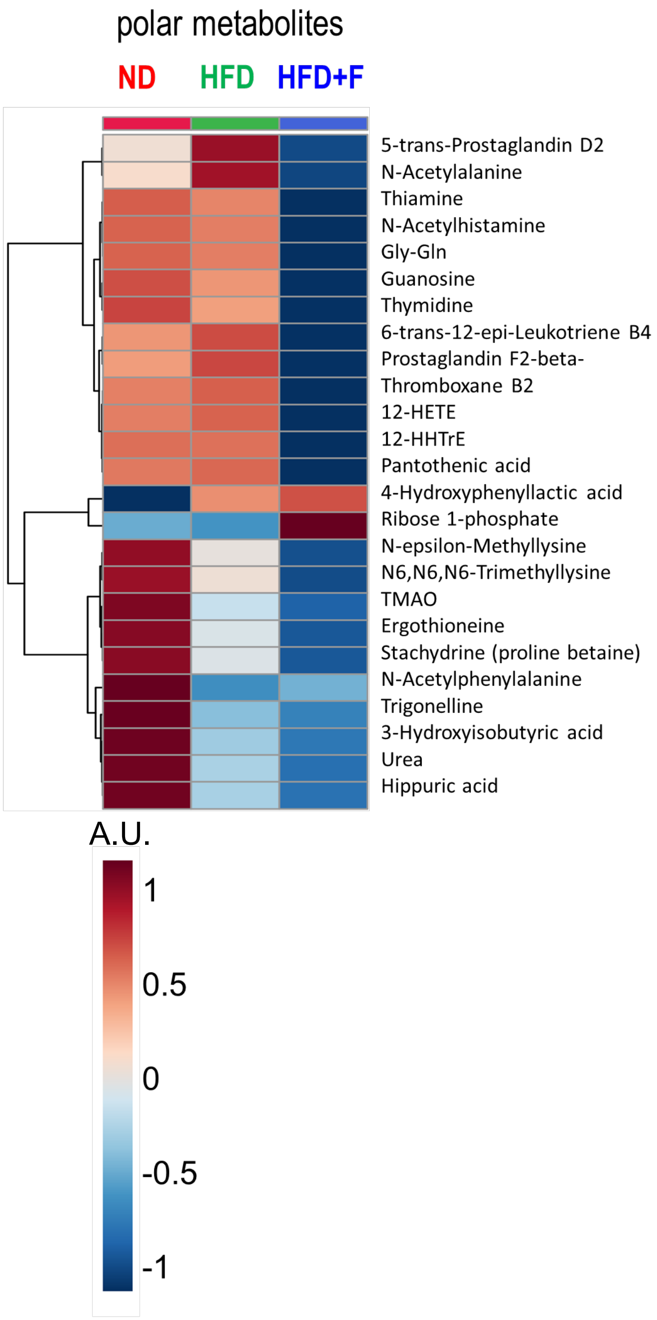

Supplementary Fig. 6 legend:
Metabolomic analysis of bone powder (BP) samples obtained from investigated mice. Heatmap of top-60 individual lipid species and top-25 polar metablites in BP (n=6 per group) based on ANOVA with p(FDR) < 0.05. (gradient color keys used in normalized intensity (A.U.)).

Supplementary Fig. 7

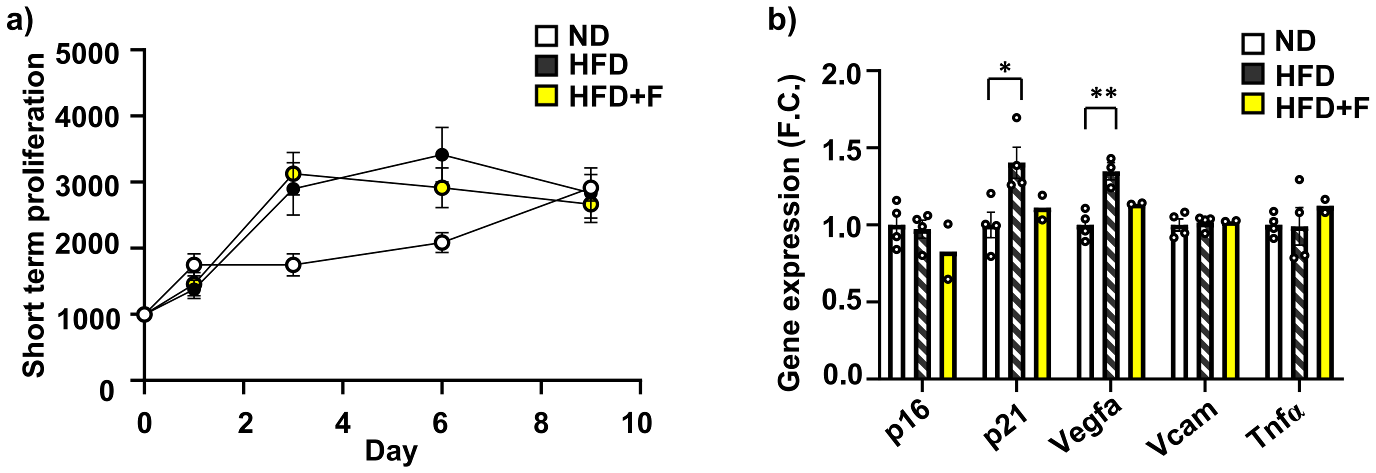

Supplementary Fig. 7 legend: Cellular characteristics of bone cells obtained from the treated mice. (**a**) Short-term proliferation assay of primary BMSCs calculated after 1, 3, 6 and 9 days in culture after seeding (n = 3 per group) (groups coding: white circle-ND, black circle-HFD, yellow circle-HFD+F). (**b**) Gene expression of senescence genes (*p16, p21, Vegfa, Vecam, Tnf*α) measured in mouse HSCs. Data are presented as mean ± SEM (n = 4 per group); one-way ANOVA, Tukey's multiple comparison test, * p ≤ 0.05, ** p ≤ 0.01. (groups coding: white column-ND, black line shading-HFD, yellow column-HFD+F)

Supplementary Fig. 8

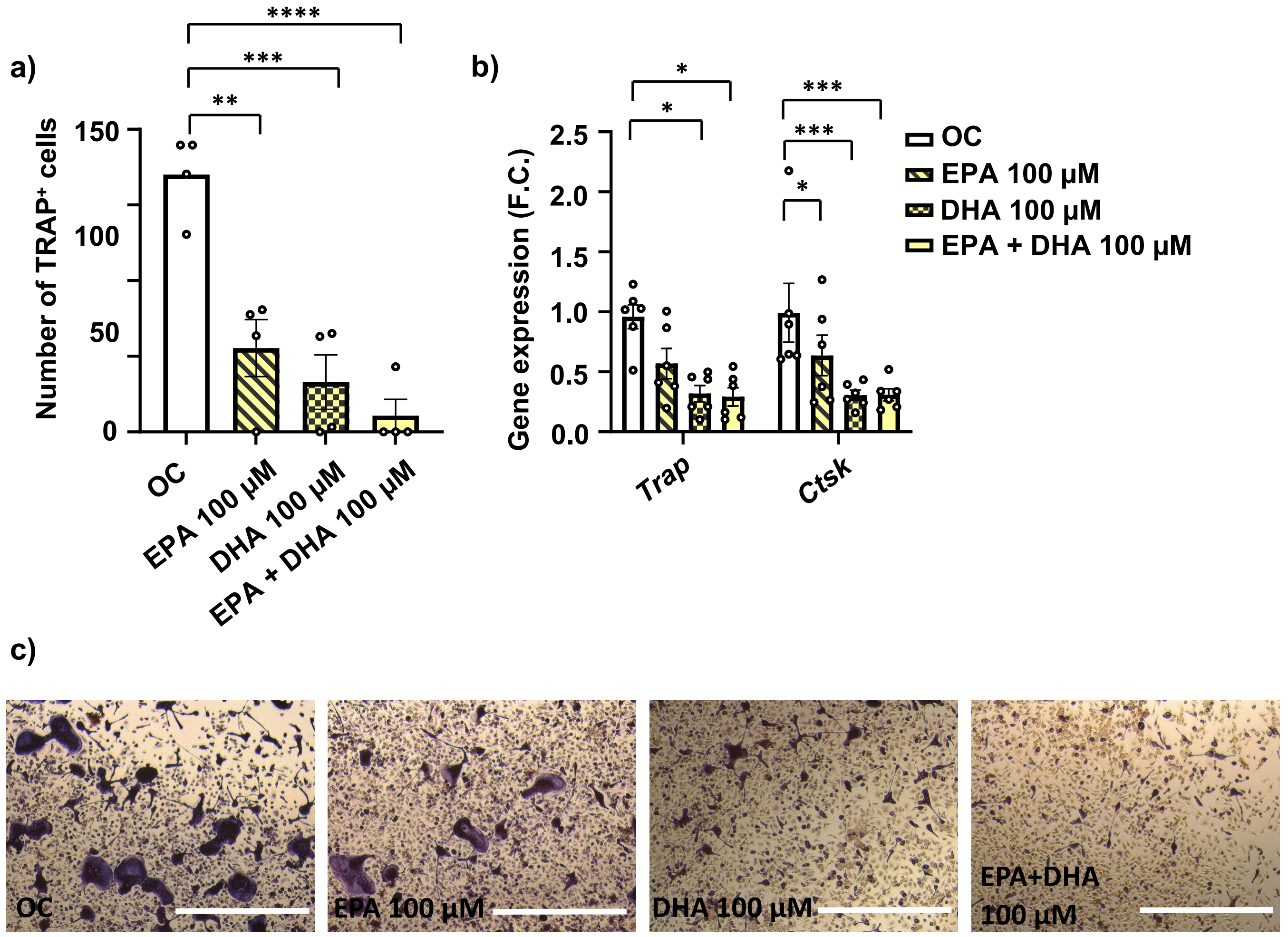

Supplementary Fig. 8 legend: The effect of omega-3 PUFA treatment in vitro on osteoclast differentiation

(**a**) Number of TRAP+ cells after 5 days of differentiation with omega-3 PUFA short term supplementation (n = 4 per group). (**b**) Gene expression of osteoclastic genes (*Trap, Ctsk*) measured in Ocs after 5 days differentiation. Data are presented as mean ± SEM (n = 6 per group); one-way ANOVA, Tukey's multiple comparison test, **p ≤ 0.01, *** p ≤ 0.001, ****p ≤ 0.0001. (**c**) Representative pictures of TRAP+ differentiated OCs after 5 days of differentiation with omega-3 PUFAs (scale bar 250 µm). (groups coding: white column-ND, black line shading-HFD, yellow column-HFD+F)

Supplementary Fig. 9

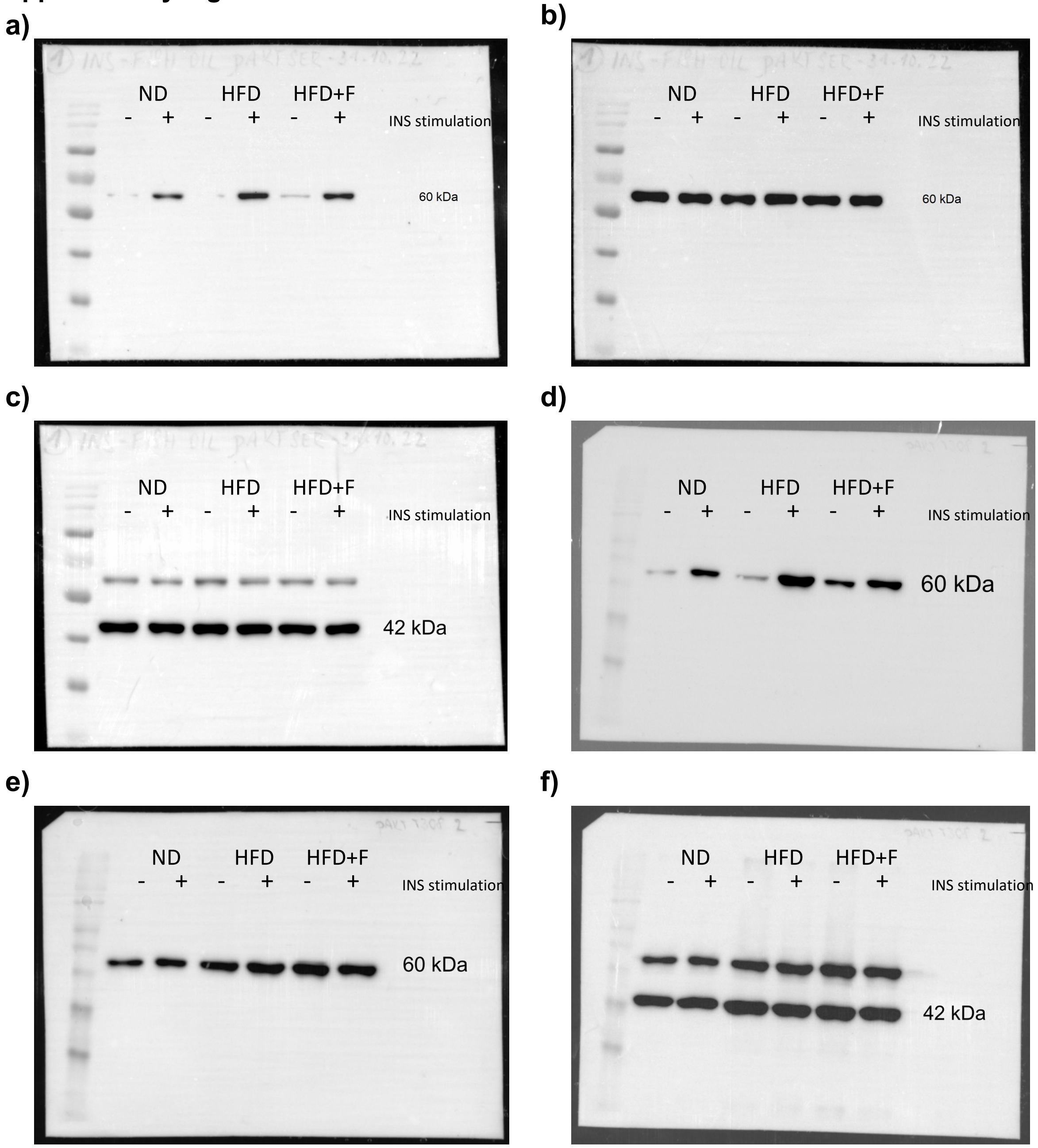

Supplementary Fig. 9 legend: Uncropped western blot membrane images corresponding to Fig.6b and Fig. 6d

(**a**) p-S473-AKT, (**b**) total AKT and corresponding (**c**) β-actin. (**e**) p-T308-AKT, (**f**) total AKT and corresponding (**g**) β-actin.

Supplementary Fig. 10

a)

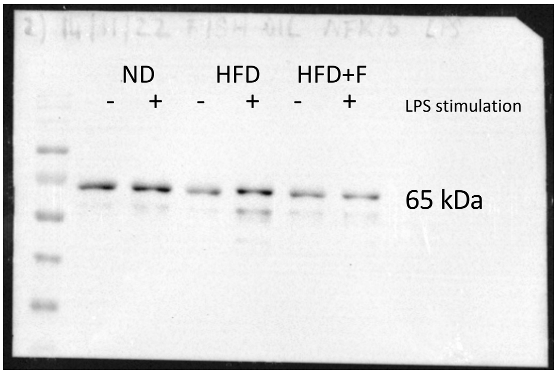

b)

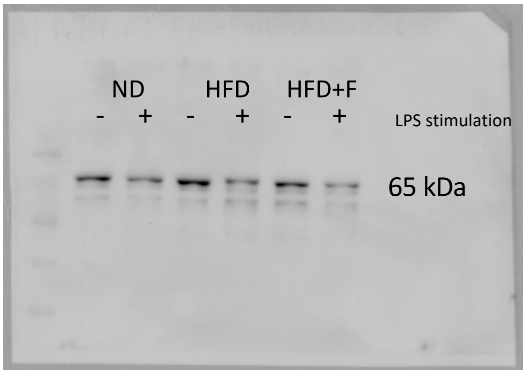

c)

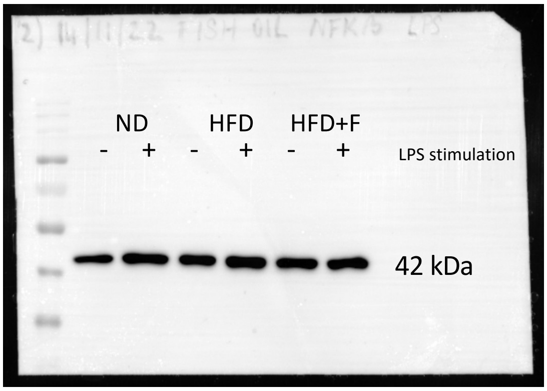

Supplementary Fig. 10 legend: Uncropped western blot membrane images corresponding to Fig. 6f. (**a**) p-NFκB, (**b**) total NFκB and corresponding (**c**) β-actin.
