# Supplementary material for: Omega-3 PUFAs prevent bone impairment and bone marrow adiposity in mouse model of obesity
Source: Commun Biol. 2023 Oct 14;6:1043. doi: 10.1038/s42003-023-05407-8 (PMC10575870; doi:10.1038/s42003-023-05407-8)
Supplement: Supplementary file 5 — Reporting Summary [file 42003_2023_5407_MOESM5_ESM.pdf]

nature portfolio

Corresponding author(s): Michaela Tencerova

Last updated by author(s): Sep 29, 2023

# Reporting Summary

Nature Portfolio wishes to improve the reproducibility of the work that we publish. This form provides structure for consistency and transparency in reporting. For further information on Nature Portfolio policies, see our Editorial Policies and the Editorial Policy Checklist.

## Statistics

For all statistical analyses, confirm that the following items are present in the figure legend, table legend, main text, or Methods section.

| n/a | Confirmed | |
|---|---|---|
| ☐ | ☒ | The exact sample size (*n*) for each experimental group/condition, given as a discrete number and unit of measurement |
| ☐ | ☒ | A statement on whether measurements were taken from distinct samples or whether the same sample was measured repeatedly |
| ☐ | ☒ | The statistical test(s) used AND whether they are one- or two-sided<br>*Only common tests should be described solely by name; describe more complex techniques in the Methods section.* |
| ☒ | ☐ | A description of all covariates tested |
| ☒ | ☐ | A description of any assumptions or corrections, such as tests of normality and adjustment for multiple comparisons |
| ☒ | ☐ | A full description of the statistical parameters including central tendency (e.g. means) or other basic estimates (e.g. regression coefficient) AND variation (e.g. standard deviation) or associated estimates of uncertainty (e.g. confidence intervals) |
| ☐ | ☒ | For null hypothesis testing, the test statistic (e.g. *F*, *t*, *r*) with confidence intervals, effect sizes, degrees of freedom and *P* value noted<br>*Give P values as exact values whenever suitable.* |
| ☒ | ☐ | For Bayesian analysis, information on the choice of priors and Markov chain Monte Carlo settings |
| ☒ | ☐ | For hierarchical and complex designs, identification of the appropriate level for tests and full reporting of outcomes |
| ☒ | ☐ | Estimates of effect sizes (e.g. Cohen's *d*, Pearson's *r*), indicating how they were calculated |

*Our web collection on statistics for biologists contains articles on many of the points above.*

## Software and code

Policy information about availability of computer code

| | |
|---|---|
| Data collection | no software was used |
| Data analysis | *Provide a description of all commercial, open source and custom code used to analyse the data in this study, specifying the version used OR state that no software was used.* |

For manuscripts utilizing custom algorithms or software that are central to the research but not yet described in published literature, software must be made available to editors and reviewers. We strongly encourage code deposition in a community repository (e.g. GitHub). See the Nature Portfolio guidelines for submitting code & software for further information.

## Data

Policy information about availability of data

All manuscripts must include a data availability statement. This statement should provide the following information, where applicable:

- Accession codes, unique identifiers, or web links for publicly available datasets
- A description of any restrictions on data availability
- For clinical datasets or third party data, please ensure that the statement adheres to our policy

Source data for the graphs and charts are available as Supplementary Data 1 and any remaining information can be obtained from the corresponding author upon reasonable request. Supplementary Material accompanies the manuscript.

## Human research participants

Policy information about studies involving human research participants and Sex and Gender in Research.

| | |
|---|---|
| Reporting on sex and gender | N/A |
| Population characteristics | N/A |
| Recruitment | N/A |
| Ethics oversight | N/A |

Note that full information on the approval of the study protocol must also be provided in the manuscript.

# Field-specific reporting

Please select the one below that is the best fit for your research. If you are not sure, read the appropriate sections before making your selection.

☒ Life sciences ☐ Behavioural & social sciences ☐ Ecological, evolutionary & environmental sciences

For a reference copy of the document with all sections, see nature.com/documents/nr-reporting-summary-flat.pdf

# Life sciences study design

All studies must disclose on these points even when the disclosure is negative.

| | |
|---|---|
| Sample size | Based on our previous study https://www.sciencedirect.com/science/article/pii/S2212877822001673, we use for the bone analysis at least 10 mice per group to reach statistical significance, to get samples for different methods and analysis- there is a repetition (2-3x) of the experiments with the same number of animals. |
| Data exclusions | The excluded values were chosen based on the values 3 times higher or lower than standard deviation. |
| Replication | we repeated on several cohorts the effect of omega-3 PUFA treatment on metabolic parameters which were confirmed in several cohorts. |
| Randomization | the mice were randomly divided into three groups in the study |
| Blinding | we did not perform blinding in this study. |

# Reporting for specific materials, systems and methods

We require information from authors about some types of materials, experimental systems and methods used in many studies. Here, indicate whether each material, system or method listed is relevant to your study. If you are not sure if a list item applies to your research, read the appropriate section before selecting a response.

## Materials & experimental systems

| n/a | Involved in the study | |
|---|---|---|
| ☐ | ☒ | Antibodies |
| ☒ | ☐ | Eukaryotic cell lines |
| ☒ | ☐ | Palaeontology and archaeology |
| ☐ | ☒ | Animals and other organisms |
| ☒ | ☐ | Clinical data |
| ☒ | ☐ | Dual use research of concern |

## Methods

| n/a | Involved in the study | |
|---|---|---|
| ☒ | ☐ | ChIP-seq |
| ☒ | ☐ | Flow cytometry |
| ☒ | ☐ | MRI-based neuroimaging |

## Antibodies

Antibodies used

Primary antibodies Catalog # Company
Phospho-Akt Ser473_ Rabbit #4058 (193H12) Cell Signaling
Phospho-Akt Thr308_Rabbit #13038 (D25E6) Cell Signaling
Total AKT_Rabbit #9272 Cell Signaling
β-actin_Rabbit #4970 (13E5) Cell Signaling
Phospho-NF-kB p65_Rabbit #3031 Cell Signaling

| | |
|---|---|
| | NF-kB p65_Rabbit #8242 (D14E12) Cell Signaling<br>Secondary HRP-conjugated antibodies<br>Anti-rabbit IgG, HRP-linked Antibody #7074 Cell Signaling |
| Validation | we validated antibodies using control cell extracts for specific proteins. |

## Animals and other research organisms

Policy information about studies involving animals; ARRIVE guidelines recommended for reporting animal research, and Sex and Gender in Research

| | |
|---|---|
| Laboratory animals | Male C57BL/6N mice (Charles River Laboratories, Sulzfeld, Germany), 12 weeks old |
| Wild animals | N/A |
| Reporting on sex | only one males, we plan to perform the studies on females as well- it is mentioned in the limitations of the study. |
| Field-collected samples | The stiudy did not involve collected samples from the field. |
| Ethics oversight | All experiments were performed according to the Institute of Physiology of the Czech Academy of Sciences guidelines and were approved under protocol 81/2016. |

Note that full information on the approval of the study protocol must also be provided in the manuscript.
